# Wellbuilt for wellbeing: Controlling relative humidity in the workplace matters for our health

**DOI:** 10.1111/ina.12618

**Published:** 2019-11-25

**Authors:** Javad Razjouyan, Hyoki Lee, Brian Gilligan, Casey Lindberg, Hung Nguyen, Kelli Canada, Alex Burton, Amir Sharafkhaneh, Karthik Srinivasan, Faiz Currim, Sudha Ram, Matthias R. Mehl, Nicole Goebel, Melisa Lunden, Seema Bhangar, Judith Heerwagen, Kevin Kampschroer, Esther M. Sternberg, Bijan Najafi

**Affiliations:** ^1^ Interdisciplinary Consortium on Ambulatory Motion Performance (iCAMP) Michael E. DeBakey Department of Surgery Baylor College of Medicine Houston TX USA; ^2^ Michael E. DeBakey VA Medical Center Houston TX USA; ^3^ U.S. General Services Administration Washington DC USA; ^4^ College of Architecture, Planning and Landscape Architecture UArizona Institute on Place, Wellbeing & Performance University of Arizona Tucson AZ USA; ^5^ HKS, Inc. Dallas TX USA; ^6^ LMI, Tysons VA USA; ^7^ Department of Biomedical Engineering University of Arizona Tucson AZ USA; ^8^ Pulmonary, Critical Medicine and Sleep Medicine Department of Medicine Baylor College of Medicine Houston TX USA; ^9^ Center for Business Intelligence and Analytics University of Arizona Tucson AZ USA; ^10^ Department of Psychology University of Arizona Tucson AZ USA; ^11^ Aclima Inc. San Francisco CA USA; ^12^ Seema Bhangar Consulting, Inc. Oakland CA USA; ^13^ University of Arizona Institute on Place, Wellbeing & Performance Andrew Weil Center for Integrative Medicine University of Arizona Tucson AZ USA

**Keywords:** health, office workers, relative humidity, sleep quality, stress responses, wearable sensors

## Abstract

This study offers a new perspective on the role of relative humidity in strategies to improve the health and wellbeing of office workers. A lack of studies of sufficient participant size and diversity relating relative humidity (RH) to measured health outcomes has been a driving factor in relaxing thermal comfort standards for RH and removing a lower limit for dry air. We examined the association between RH and objectively measured stress responses, physical activity (PA), and sleep quality. A diverse group of office workers (n = 134) from four well‐functioning federal buildings wore chest‐mounted heart rate variability monitors for three consecutive days, while at the same time, RH and temperature (*T*) were measured in their workplaces. Those who spent the majority of their time at the office in conditions of 30%‐60% RH experienced 25% less stress at the office than those who spent the majority of their time in drier conditions. Further, a correlational study of our stress response suggests optimal values for RH may exist within an even narrower range around 45%. Finally, we found an indirect effect of objectively measured poorer sleep quality, mediated by stress responses, for those outside this range.

**Practical Implications**

- Thermal comfort standards for relative humidity (RH) in buildings have been relaxed over the past 30 years and since ASHRAE 55‐1989 there has been no lower limit to RH in thermal comfort or ventilation standards.
- This project is the first to study associations between RH and three objectively measured health metrics including stress responses (heart rate variability), sleep quality, and physical activity in real time in a large observational study conducted in office buildings.
- We found that individuals who spent the majority of measured time within the range set in ASHRAE 55‐1989 (30%‐60% RH) experienced lower stress responses at the office and better sleep quality than those who did not.
- Our results support further research to parse potential direct versus indirect effects, confounders, and to establish a mechanistic link between RH and health outcomes.

## 1 INTRODUCTION

The US General Services Administration's Wellbuilt for Wellbeing research project, led by the University of Arizona's Institute on Place, Wellbeing & Performance, was an exploratory study investigating how health‐related metrics change as measured levels of indoor environmental quality (IEQ) factors change in real time. Using a cross‐sectional, observational study design, we saw a strong correlation between changes in relative humidity (RH) and changes in stress response. Specifically, we measured a 25% difference in stress response levels between those spending the majority of their time in the 30%‐60% RH range set in ASHRAE 55‐1989 and those in drier conditions. The difference is of moderate effect size and may represent clinically significant levels cumulatively, over long‐term exposure.^1^, ^2^ This is important because Americans spend more than 90% of their time indoors and over 50 million US office workers spend 20% of their time at the office.^3^, ^4^ Policymakers, standards developers, and building design and operations professionals are increasingly interested in indoor environment quality (IEQ) and its effect on occupant health and comfort. Despite several studies revealing the potential negative impact of both too high and too low relative humidity (RH) on health and comfort,^5^, ^6^, ^7^, ^8^, ^9^, ^10^ the importance of controlling RH has received less attention than other IEQ parameters and lower limits have been removed from thermal comfort standards.

There is an ongoing debate about the influence of RH on perceived indoor air quality and comfort and the impact RH has on health. The debate involves whether (a) RH has little or no direct effect on health or comfort, which are instead driven by the presence of indoor pollutants; (b) RH has a mediating effect caused by impacts to the precorneal tear film and mucosal membranes in the eyes, skin and airways, which make individuals more susceptible to the effects of pollutants; or (c) RH has a direct effect on perceived health and comfort. Several studies illustrate the extremes of this debate. A chamber study of 8 healthy, male, college‐aged students found no impact of relative humidity,^11^ while other studies concluded that indoor pollutants such as VOCs emitted from building materials and or particulates were the likely cause of perceived “dry air” and irritation of skin, eyes, and airways.^12^, ^13^, ^14^ Furthermore, humans lack sensory receptors for humidity^5^, ^13^, ^14^, ^15^, ^16^ and perceive changes in RH indirectly through other sensory pathways which may affect the influence it has on perceived thermal comfort. Other literature finds plausible connections between RH levels and risk factors for eye irritation and sleep quality,^7^ production of cortisol in the skin,^15^ and the transmission, survivability, and virility of influenza viruses.^6^, ^8^ These contrasting findings indicate that the relationship between perceived comfort, health outcomes and humidity is complex and warrants further study.

This complexity and lack of multidisciplinary, well‐controlled, field‐based study has affected how RH is treated in building codes and standards. Relative humidity levels in buildings are influenced by occupancy, ambient climate, and building design, construction, and operation. An earlier version of the most commonly used US standard for indoor air quality, ASHRAE Standard 55 (ASHRAE 55‐1989), required 30%‐60% RH in office buildings for thermal comfort.^16^ That standard was later relaxed by eliminating the lower RH limit, when ASHRAE began positioning its standards as a basis for building codes that focused on low energy consumption. ASHRAE found insufficient evidence based on real‐world conditions to support negative impacts on wellbeing.^17^ Earlier research by Sterling et al^18^ suggested that a mid‐range value of 40%‐60% RH might be optimal for prevention of a number of health issues in buildings. However, while Derby et al^6^ reiterated many of Sterling's findings, they caution against mandating a general, lower limit without additional research involving a larger number of subjects, a more diverse study population, and conducted in real‐world settings. Additionally, prior research relating RH to health outcome metrics has considered just one outcome factor belying the complex nature of any potential relationship.^19^, ^20^, ^21^

Our Wellbuilt for Wellbeing study is novel and important to the debate for three reasons. First, we recorded multiple objectively measured health‐related metrics using wearable devices (eg, stress responses, sleep quality, and physical activity) where most prior work has focused on only one. Second, our dataset is diverse, derived from the participation of 134 individuals of mixed age, gender and occupations from 4 different buildings over a 2‐year study period. Third, we collected tens of thousands of 5‐min observations of RH and health metrics together, in the field, in real time. Our health metrics lend important new perspective on the relationship between RH and health. There is evidence that persistent exposure to conditions that result in short‐term stress responses contributes to allostatic loading**,** or the physical wear and tear on the body caused by repeated or chronic stress over time, which may increase the odds of long‐term health impacts.^1^, ^2^ Additionally, the health metrics have been shown in past research to form a reciprocal relationship indicating a possible indirect relationship.^22^, ^23^ Based on our review of extant literature, we expected the health‐related metrics to degrade as conditions became drier or more humid than in the middle range of 30%‐60% RH established in the ASHRAE 55‐1989 thermal comfort standard.

## 2 METHODS

### 2.1 Participants and setting

Our observational study investigated the influence of indoor environmental quality (IEQ) factors on human health. Participants were office workers who performed a variety of office‐based roles for the federal government in the mid‐Atlantic and southcentral regions of the United States. The offices selected represent different common workstation layouts and designs from across the US General Services Administration's portfolio of office space, which accommodates over 1 million federal employees. The study collected data between May 5, 2015, and August 25, 2016. After signing a consent form, the participants enrolled in the study and then underwent an orientation session regarding the study and the sensors involved. Participants completed several questionnaires on demographics, medical history, and the Pittsburgh Sleep Quality Index (PSQI), as well as measures of perceived stress and comfort. At the end of the orientation session, participants were instructed to wear a chest sensor monitoring heart and physical activity for three consecutive work days and two nights.

Inclusion criteria included anyone who was ambulatory (able to walk for 20 m without aid), working in one of the designated federal office buildings, and present in the office during work time for three consecutive days. A research coordinator recorded the start and end of each participant's work day. Time outside the office began one hour after leaving the office and ended one hour before the onset of sleep. Exclusion criteria included those who were pregnant or using a pacemaker or insulin pump. The study was approved by the University of Arizona Institutional Review Board (IRB #502692356A006).

### 2.2 Cardiac activity measurement (stress responses)

To record cardiac activity, we used a chest‐worn sensor, EcgMove 3 (movisens GmbH, Karlsruhe, Germany). On the basis of participants’ preference, the sensor was placed below the sternum with two standard electrocardiography patches attached to either the skin or on a chest belt.^24^ To quantify stress responses, we quantified heart rate variability by two standard time‐dependent measures including the mean of standard deviations (SDNN) and the square root of the mean squared differences (RMSSD) for all successive normalized‐to‐normalized heart rate intervals.^25^ SDNN is a global index of HRV and reflects longer‐term circulation differences and circadian rhythm. Lower SDNN values indicate higher stress responses, and higher SDNN values are linked to better wellbeing.^26^ RMSSD is another method to quantify heart rate variability, which reflects vagus tone. Higher values equate to higher parasympathetic activities or more relaxation.^25^ We calculated both SDNN and RMSSD in every 5‐minute interval according to the guidelines of the European Society of Cardiology and the North American Society of Pacing and Electrophysiology.^25^ We observed a very high interclass correlation (ICC) for SDNN (single measure = 0.849 (0.810, 0.882); average measure = 0.944 (0.928, 0.957)). The same scenario observed for RMSSD (single measure = 0.852 (0.814, 0.885); average measure = 0.945 (0.929, 0.958)). We then took the average of these across the entire interval of interest (ie, at the office, outside the office, and during time in bed). To simplify the visual presentation of these data, we reverse the scale for SDNN and RMSSD in our figures presenting the data as 100 ms—any reported value.

### 2.3 Physical activity measurement

The EcgMove 3 sensor includes a tri‐axial accelerometer, which quantified physical activities using validated algorithms described in previous studies.^27^, ^28^, ^29^ We assessed a number of physical activity metrics at the office including activity postures (sitting, standing, walking, lying), postural transitions (sit‐to‐stand, sit‐to‐lie, stand‐to‐walk), total number of taken steps, average of unbroken walking bouts in seconds (average duration of all recorded continuous walking bouts with a minimum of three consecutive steps), and average physical activity level (defined by the intensity of the acceleration magnitude [mG] per second). This last measure is our main method of capturing overall physical activity as it can be connected to clinical definitions for sedentary behavior and was utilized in previous work by our Wellbuilt for Wellbeing project.^30^ To capture physical activity outside of the office, we estimated the percentage of activity behavior (eg, sedentary, light, and moderate‐to‐vigorous activities) using a validated algorithm tailored for this study.^31^

### 2.4 Sleep quality measurement

Sleep quality was objectively measured from chest‐worn sensor data relating to the motion, position, and posture of the participant's torso while in bed. We used a composite score called the sensor‐based sleep quality index (SB‐SQI).^32^ SB‐SQI is based on a validated algorithm estimating sleep‐onset latency, total sleep time, and sleep efficiency from the chest‐worn sensor data^27^ and then applying the scoring method from the Pittsburg Sleep Quality^33^ to present sleep quality. Although organized in similar fashion to the PSQI, the inputs for SB‐SQI scoring are objective measures derived from sensor data.

### 2.5 Relative humidity (RH) and other IEQ measurement

We took continuous measurements of ambient levels of several IEQ factors at the workplace including RH,^34^ T, carbon dioxide (CO2), and particulate matter (PM) as previously reported^35^ (Figures S15–S18). Measurements were made using an Aclima (Aclima Inc) measurement platform consisting of individual sensing nodes mounted on the walls near participants. The 1 Hz time‐series RH data were transmitted via Wi‐Fi or Ethernet to cloud‐based servers where the data are processed, analyzed, and stored. To evaluate levels of RH exposure, we tracked participants’ proximity to ambient RH measures using calendar information, recorded logs, and floor plan coding.^35^ The RH sensor reports values that range from 10% to 90%, with a resolution of 0.3% and an accuracy of ±4%. Additional descriptions of sensors including type, range, resolution, and accuracy are included in supplemental materials S15–S18.

**Figure 1:**
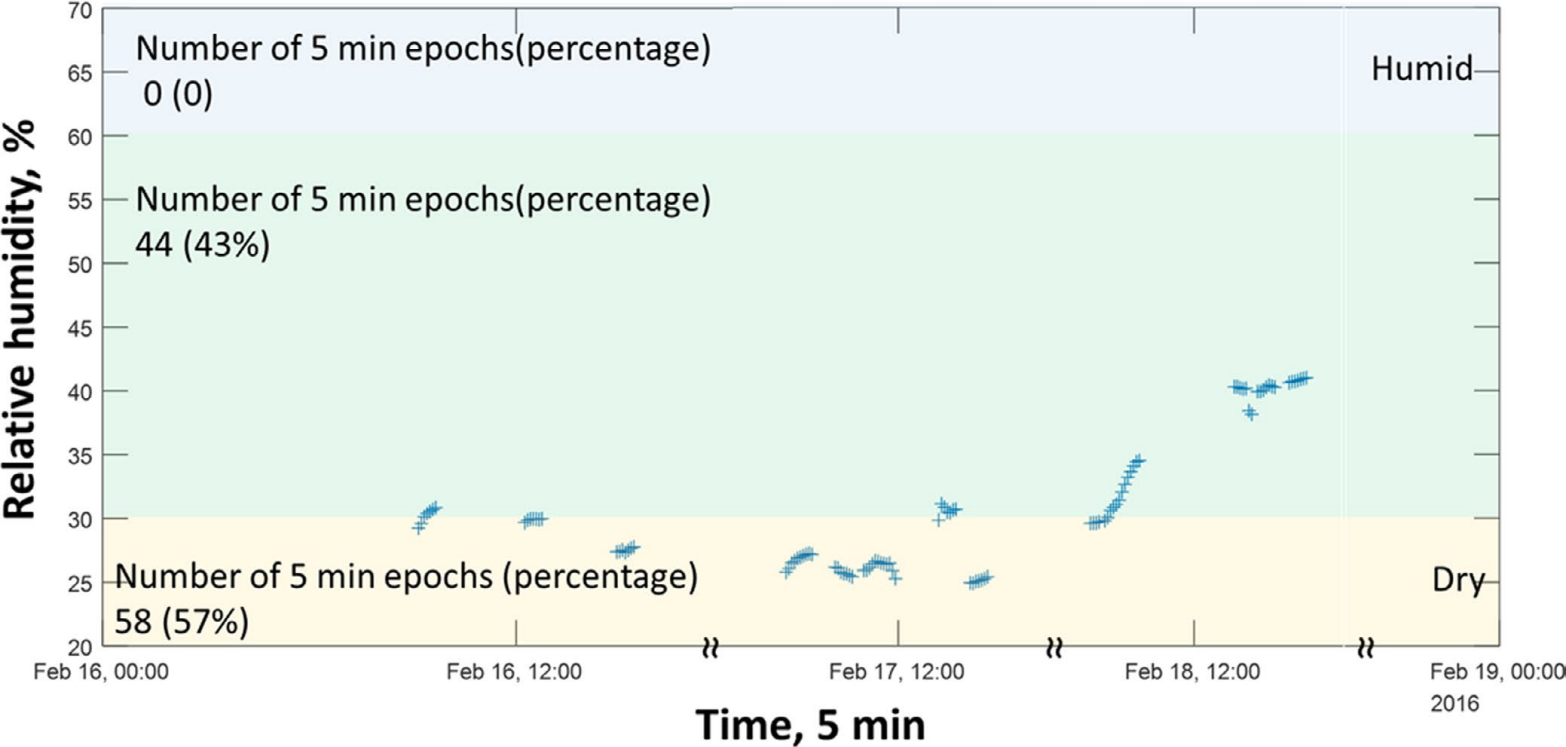
An illustration of recorded relative humidity (RH) over three days of recording for an individual participant. We matched the location of the participant, when available, to the relevant RH measurements from the environmental sensor in the proximity of the participant. 57% of this participant's data were recorded below 30% RH and they were therefore grouped in the “dry” category

The participants’ recorded IEQ data were averaged every 5 minutes, resulting in a time‐series of RH exposures for each participant over the course of their time at the office (Figure 1). We then classified participants based on whether more than 50% of their measured RH values were in or out of the 30%‐60% comfort range established by ASHRAE 55‐1989.^18^ This resulted in 3 groups:

- 1 = dry: majority of measurements < 30% RH,
- 2 = comfort‐humidity: majority of measurements ≥ 30% and ≤ 60% RH, and
- 3 = humid: majority of measurements > 60% RH

### 2.6 Statistical analysis

We performed all statistical analysis using SPSS statistics 23.0 (IBM), except the structural equation modeling (SEM), which was performed with R statistical package version 3.4.1 and the lavaan package.^36^ We used independent *t* tests (proportional) or chi‐square tests (bivariate), as appropriate, to compare the demographic information of groups. Univariate analysis compared group differences for investigated variables with age, BMI, and seasonal effect as covariates. We measured effect size (*ES*) as Cohen's *d* with a range of small (~0.02), medium (~0.5), and large (~0.8).^37^ We used several structural equation models (SEM) to estimate the direct and indirect effects of RH on outcome measures. In these models, we selected SDNN for representation of the stress responses, the SB‐SQI score to represent sleep quality, and the general tri‐axial accelerometer data (mG) to represent average physical activity at the office. We explored the idea that exposure to different levels of RH could affect one's stress responses as well as physical activity at the office, which in turn could lead to differences in sleep quality as anticipated by the reciprocal relationship between these health‐related metrics.^22^, ^23^, ^35^ Therefore, for structural equation modeling and ease of interpretation of the association between sleep and average physical activity and RH mediated by stress, SDNN values were reversed and multiplied by a constant value of 0.01. Global estimation with maximum‐likelihood approach^38^ simultaneously estimated the path coefficients, while bootstrapping derived the standard error of estimates. We handled missing values in the model using the full maximum‐likelihood method.^39^ We tested the sensitivity of each variable using analysis of variance (ANOVA)/linear regression as well as a comparison of modification indexes.^40^

#### 2.6.1 Correlational analysis

We evaluated the relationship between noncategorized RH exposure and stress responses using a series of exploratory correlations. This exploratory work is similar to previous analyses described in quantum correlations and synchronization measures.^41^ The correlation was measured by the correlation coefficient, which represents the strength of the linear relationship between the stress responses and RH exposures at each comparison point as the following:

(1) $$r_{K}=\frac{\sum_{i=1}^{n}\left(x_{i,K}-{\bar{x}}_{K}\right)\left(y_{i}-\bar{y}\right)}{\sqrt{\sum_{i=1}^{n}{\left(x_{i,K}-{\bar{x}}_{K}\right)}^{2}}\sqrt{\sum_{i=1}^{n}{\left(y_{i,}-\bar{y}\right)}^{2}}}$$

where *r* is a correlation coefficient, *x* is a RH value converted by a RH comparison point, and *y* is the stress response defined by averaged SDNN of each participant. The RH comparison point ranged from 30% to 60% at 1% increments as the following:

(2) $$x_{K}=\left|K-h\right|,\left(K=30,31,32,\ldots ,59,60\right)$$

where *K* is the humidity comparison point and *h* is a measured relative humidity. For example, to get the correlation coefficient for the RH comparison point of 30%, the measured RH was subtracted by 30 then the absolute value was calculated so that a range of the converted RH value is from 0 to 70. Then, the converted RH values were applied to the above equation to get the correlation coefficient. This procedure was repeated up to the RH comparison point of 60.

## 3 RESULTS

### 3.1 Participants

**Figure 2:**
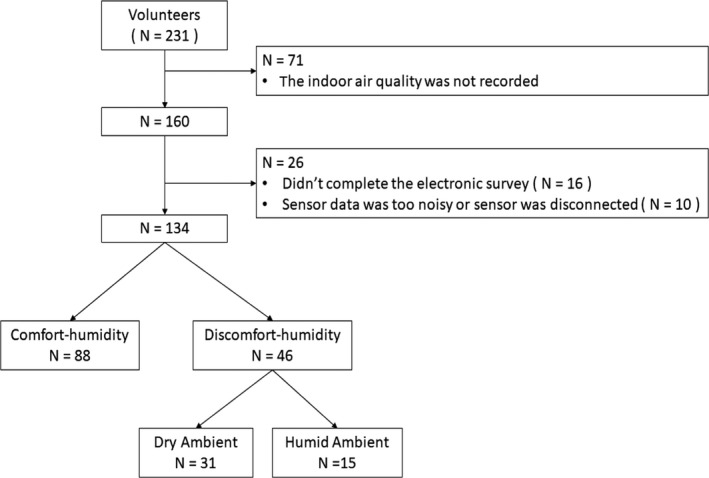
Recruitment diagram for participants

A total of 231 white‐collar office workers (54.5% female, average age 43.4 years) participated in this study. Given participant proximity to ambient RH measurement sensors, 134 individuals had sufficient data for analysis. For our analyses, sufficient data were considered to be at least 40 minutes of measured RH data (this quantity exceeds minimum timeframe necessary to assess the effect of RH on heart rate).^21^ Table 1 summarizes the demographic, clinical, and sensor‐derived parameters, and the schema of recruitment is illustrated in Figure 2. Of the 34% of workers who were in the discomfort‐humidity group, 67% (n = 31) were in the dry condition and 33% (n = 15) were in the humid condition. No differences were observed between groups for demographics (age, BMI, and sex), experience level, and years of education (Table 1).

**Table 1:** Demographics and clinical characteristics

	Comfort‐humidity zone mean (SD)	Discomfort‐humidity zone mean (SD)	*P*‐value
Number of participants	88	46	‐
Age, y	43.1 (12.9)	43.6 (10.5)	0.13
BMI, kg/cm^2^	26.5 (6.3)	27.8 (4.5)	0.66
Obesity (BMI ≥ 30), N (%)	21 (24%)	7 (15%)	0.20
Female sex, N (%)	40 (45%)	26 (46%)	0.17
Experience, y	10.4 (10.0)	10.7 (9.8)	0.22
Education			
High school grad or less	3	2	0.32
Some college	45	18	0.86
College+	40	26	0.86
Total recording, h	7.6 (0.8)	7.5 (1.0)	0.24
Pittsburg Sleep Quality Index	6.49 (3.11)	6.16 (3.02)	0.60
Cardio vascular problems	20 (23%)	8 (17%)	0.61
Depression	8 (10%)	5 (11%)	0.76
Frequency of back/neck pain			
No pain	20 (23%)	16 (35%)	0.31
Few times per year	24 (27%)	11 (24%)	0.32
Few times per month	18 (20%)	9 (20%)	0.52
Few times per week/constant	36 (41%)	12 (26%)	0.52
Smoking	5 (6%)	3 (6%)	

Abbreviations: BMI, body mass index; SD, standard deviation.

### 3.2 Impact of relative humidity on health‐related metrics

#### 3.2.1 Stress responses

**Figure 3:**
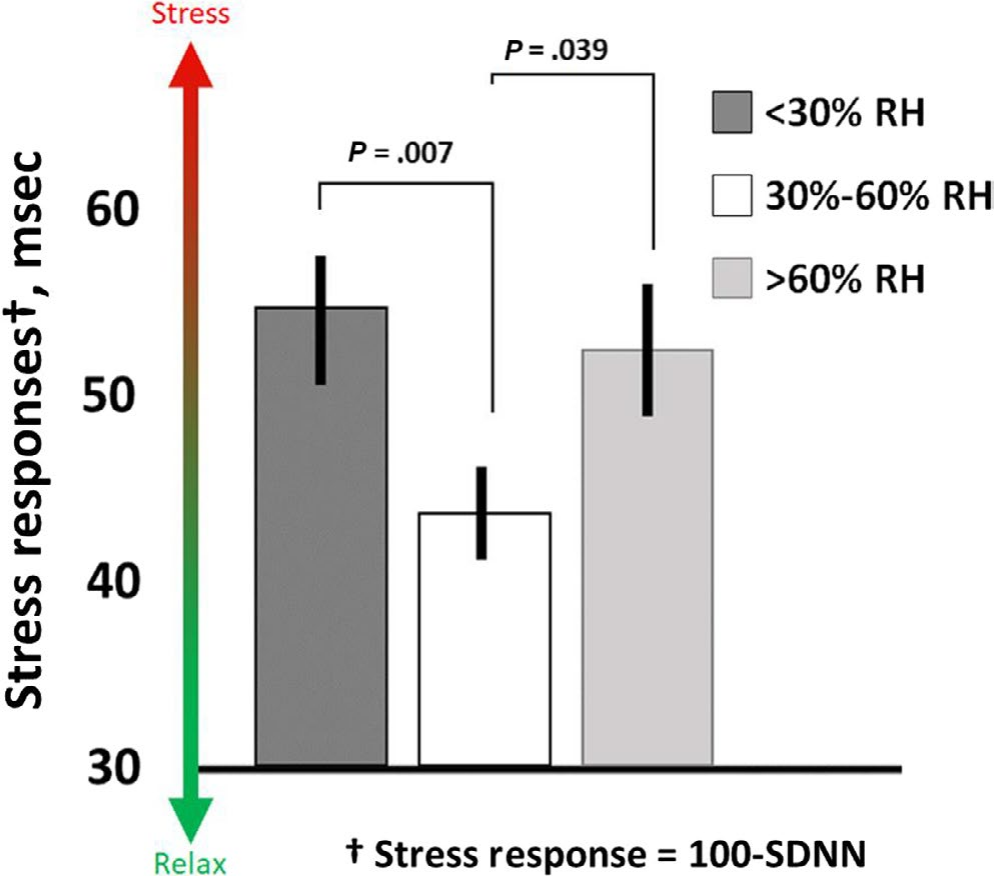
Group comparison of individuals with majority of recorded exposure within dry, comfort, and humid conditions. Those in dry and humid conditions experienced 25% and 19% more stress, respectively, than those in the comfort condition. These differences are of moderate effect size. The results for the dry and humid conditions were similar enough to collapse into a single “discomfort‐humidity” grouping for subsequent analyses. Results were adjusted by age, BMI, and season. These stress response values are presented as 100 ms – SDNN to simplify the visual representation of higher stress

Individuals in the dry and humid groups experienced 25% and 19% higher stress responses (lower SDNN), respectively, compared to those in the 30%‐60% RH group (*P* < .050, Figure 3). These differences are of moderate effect size as shown in Table 2. Because participant responses in dry and humid conditions were similar, we then merged these groups into a single “discomfort” category (ie, outside 30%‐60% RH). However, building operations typically attempt to control high humidity, and not low humidity, and the dry conditions have a stronger stress response.

**Table 2:** Between‐group comparisons for sensor‐derived parameters, including stress responses, physical activity, and sleep quality at the office, outside the office, and during time in bed

Parameters	Discomfort‐humidity mean (SD)	Comfort‐humidity mean (SD)	Difference (%)	95% CI	*P*‐value	Effect size^a^
Stress responses						
At the office						
SDNN, ms	46.28 (15.92)	56.36 (15.38)	−10.08 (−22)	−16.02 to −4.14	**.001**	0.64
RMSSD, ms	22.65 (12.07)	29.07 (11.66)	−6.41 (−28)	−10.92 to −1.91	**.006**	0.54
Outside the office						
SDNN, ms	44.94 (15.49)	50.95 (15.13)	−6.01 (−13)	−12.16 to 0.14	.06	0.39
RMSSD, ms	23.34 (12.72)	26.27 (12.42)	−2.93 (−13)	−7.98 to 2.12	.25	0.23
Time in bed						
SDNN, ms	49.47 (19.04)	56.72 (18.54)	−7.26 (−15)	−14.65 to 0.13	.05	0.39
RMSSD, ms	30.61 (19.39)	38.15 (18.88)	−7.54 (−25)	−15.06 to −0.01	**.050**	0.39
Sleep quality parameters						
Sleep onset latency, min	8.31 (4.83)	8.61 (4.71)	−0.30 (−4)	−2.17 to 1.58	.76	0.06
Total sleep time, min	354.66 (56.13)	360.32 (54.70)	−5.66 (−2)	−27.49 to 16.17	.61	0.10
Wake after sleep onset, min	66.58 (34.44)	67.64 (33.56)	−1.07 (−2)	−14.46 to 12.33	.88	0.03
Sleep efficiency, %	84.43 (7.27)	84.39 (7.08)	0.04 (0)	−2.79 to 2.86	.98	0.00
Sensor‐based sleep quality index	2.52 (1.42)	2.39 (1.38)	0.14 (5)	−0.41 to 0.69	.63	0.10
Physical activity						
At the office						
Steps	3431.13 (1692.13)	3845.96 (1640.69)	−414.83 (−12)	−1047.36 to 217.71	.20	0.25
Walk, %	6.69 (2.99)	7.62 (2.90)	−0.93 (−14)	−2.05 to 0.19	.10	0.32
Stand, %	9.47 (4.39)	8.30 (4.26)	1.17 (12)	−0.47 to 2.81	.16	0.27
Sit, %	83.84 (6.06)	84.08 (5.88)	−0.24 (0)	−2.51 to 2.03	.84	0.04
Sit, min	382.99 (61.58)	377.97 (59.71)	5.02 (1)	−18.00 to 28.04	.67	0.08
Average level of activity, mG/min	18.44 (7.62)	20.72 (7.36)	−2.27 (−12)	−5.08 to 0.53	.11	0.30
Average walking bout, sec	14.84 (5.41)	17.73 (5.24)	−2.89 (−19)	−4.92 to − 0.87	**.005**	0.54
Outside the office						
Sedentary behavior, %	74.75 (12.09)	70.15 (11.76)	4.59 (6)	−0.19 to 9.38	.06	0.39
Moderate‐to‐vigorous activity, %	5.33 (5.58)	7.73 (5.43)	−2.40 (−45)	−4.61 to −0.19	**.033**	0.44

Note

The bold text shows the significant *P*‐value <.05.

Abbreviations: 95% CI, 95 percent of confidence intervals; RMSSD, root mean squared of successive differences of N‐to‐N intervals; SDNN, standard deviation of normalized N‐to‐N intervals.

a Cohen's d effect size.

**Figure 4:**
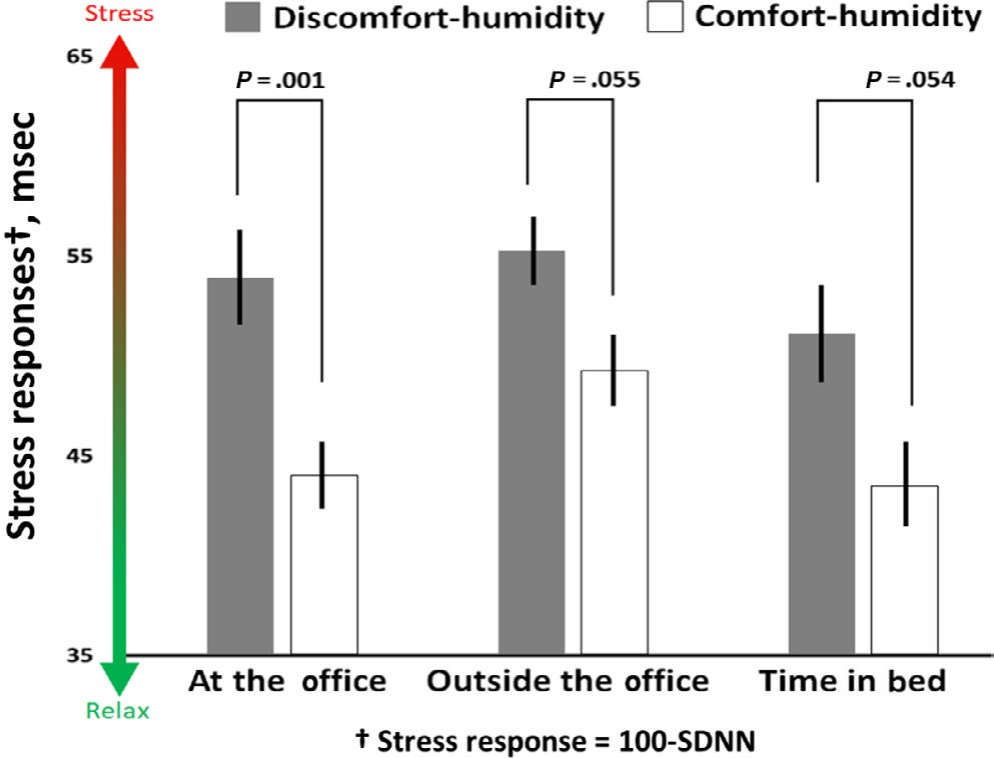
Comparison between groups with majority exposure inside or outside of the 30%‐60% relative humidity range established by ASHRAE 55‐1989 while at the office. Stress responses were quantified by a comparison of heart rate variability (SDNN) between these groups. Those in the discomfort‐humidity group at the office had lower SDNN (higher stress) in comparison with the comfort‐humidity group at the office as well as outside the office. Results were adjusted by age, BMI and season. These stress response values are presented as 100 ms—SDNN to simplify the visual representation of higher stress

On average, participants in the discomfort group experienced higher stress responses (lower SDNN) compared to the comfort group (22%, *P* = .006, ES = 0.54). This group also had higher stress responses outside of the office (13%, *P* = .055, ES = 0.39) and at night (15%, *P* = .054, ES = 0.39) though these findings were not statistically significant (Figure 4). Similar trends were observed when the metric indicator of relaxation (RMSSD) was considered (Figure S2).

#### 3.2.2 Evidence of an optimum range for relative humidity and stress responses

**Figure 5:**
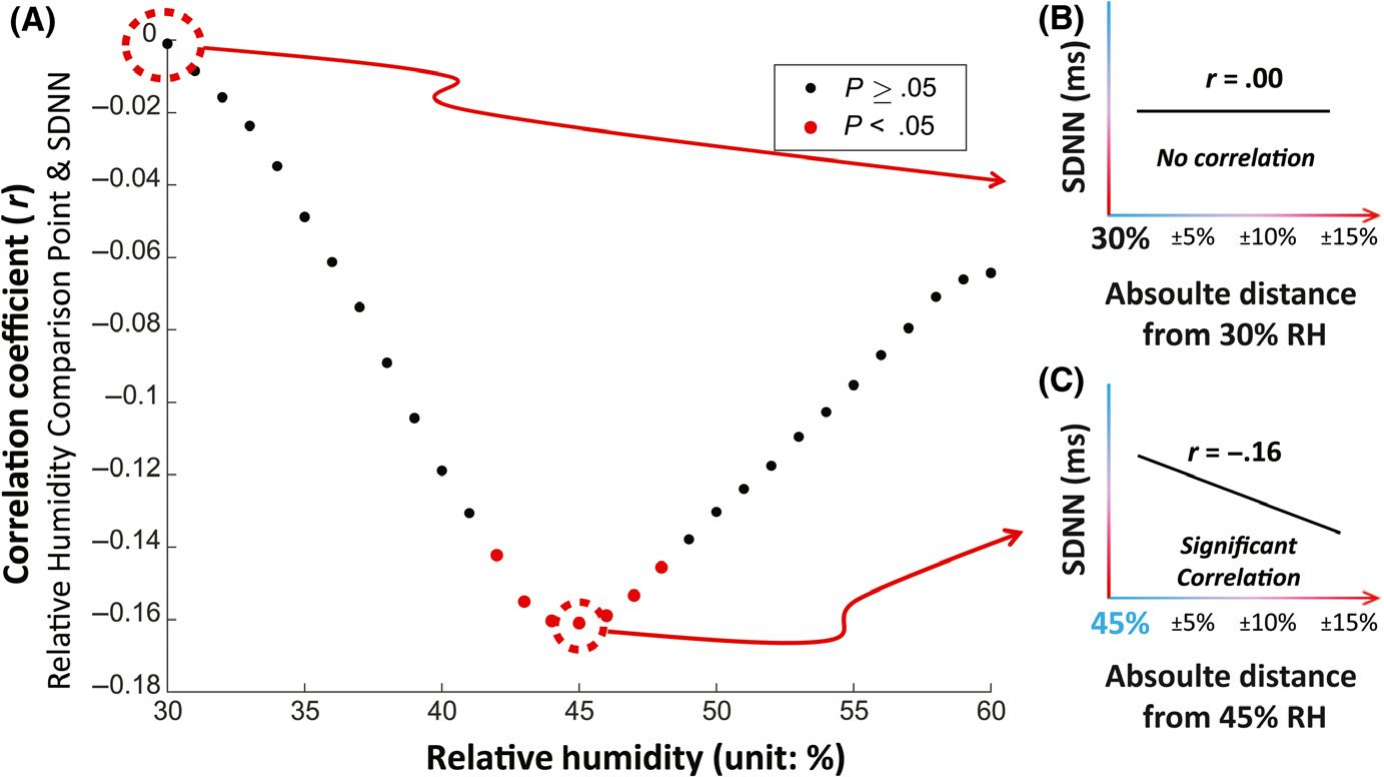
Exploratory analysis of noncategorized RH exposure and stress responses. A, We studied the correlation between participants’ average SDNN and the corresponding absolute distance between participants’ average RH exposure and each comparison point between 30% and 60% RH. We then plotted that correlation on the y‐axis at each comparison point. B, At the 30% RH comparison point, there was no correlation. C, All RH comparison points between 42% and 48% RH were statistically significant. At 45% RH, we saw the highest correlation. This suggests an optimal range in the relationship between RH and stress response, where RH values on either side of 45% (eg, drier or more humid conditions) are associated with higher stress

The correlation analysis of stress responses yielded evidence of an optimum range for the relation between relative humidity and stress responses. Correlations that are significant and negative suggest that for participants who had RH exposures farther away from the comparison point had higher stress responses (lower SDNN values) compared to participants with RH exposures closer to the comparison point. The only correlations that were significant (*P* < .050) were for comparison points between 42% and 48% RH. The comparison point of 45% RH reached a higher correlation even after adjusting for age and BMI (unadjusted r = −.16, *P* < .001; adjusted r = −.24, *P* = .001). We then plotted each correlation result as a single point in order to visually represent any patterns. Overall, this model suggests the possibility of an optimal range in the relationship between RH and stress response around 45% RH, where RH values on either side of 45% (eg, drier or more humid conditions) are associated with higher stress (Figure 5).

#### 3.2.3 Physical activity and sleep quality

We performed group comparisons for sleep quality and physical activity health‐related metrics. We did not find a significant difference in sleep quality between groups. Additionally, while we found significant differences in total step counts and duration of walking bouts, we did not find a significant difference in overall physical activity (mG). Overall physical activity was our primary health‐related metric (Table 2 and Figure S5).

#### 3.2.4 Structural equation model for stress responses, sleep quality and physical activity

**Figure 6:**
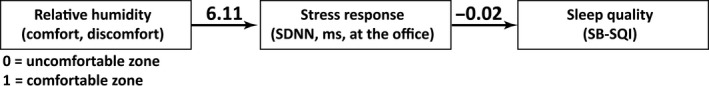
Structural equation modeling revealed a significant indirect effect of RH on sleep quality mediated by stress responses

Despite the fact that we did not observe direct effects between one's RH grouping and sleep quality (SB‐SQI), we observed a significant mediating effect for stress responses (SDNN) between the two RH groups with small effect size (B = −0.02 standard error = 0.01, 95% CI = −0.03, −0.01; Figure 6). In other words, inclusion in the discomfort humidity group was related to higher stress responses at the office, which in turn was related to lower measured sleep quality. We did not observe a similar mediating effect of physical activity (mG) for the relationship between RH grouping and sleep quality, nor was physical activity a significant mediator between RH grouping and stress responses (SDNN). We did, however, confirm the relationship between individual's RH grouping and stress responses at the office discussed above (B = 6.11, standard error = 2.23, 95% CI = 1.71, 10.51). For the full SEM model results and paths tested, see Table S6 and Figure S7.

## 4 DISCUSSION

Our study measured a 25% difference in stress response levels between those spending the majority of their time in the 30%‐60% RH range set in ASHRAE 55‐1989 and those in drier conditions. The ASHRAE standard, which has since been superseded and the lower limit removed, may be beneficial for reasons more imperative than thermal comfort—that is, for both its direct impact on stress responses and indirect correlation with sleep quality mediated by stress responses. Furthermore, we found indication that a narrower range which minimizes stress responses around 45% RH may exist. There are potential confounds to our findings and additional studies through controlled interventions are needed to provide evidence for a causal link between RH and stress. Fortunately, suggestion of a narrower range for optimal RH aligns with several immediate strategies that could both potentially improve occupant comfort and reduce energy consumption and costs associated with building operations as described later in this section.

Our study adds an important new human outcome‐based perspective to work relating RH to the health of building occupants. Specifically, it demonstrates a relationship between RH and participants’ stress responses in real‐world conditions, over a relatively long and continuous recording period, without altering participants’ work routine or daily activity. The observed relationship is of moderate effect size. To our knowledge, there is no well‐established threshold to determine clinically meaningful difference in stress response value. However, previous studies involving chronic exposure to small to moderate stressors and the concept of allostatic loading suggest it may be clinicallycsignificant cumulatively, over long‐term exposure.^1^, ^2^ Our literature review provides some basis for the increased stress response. Previous studies found connections between acute conditions such as deterioration of precorneal tear film in the eyes and mucosal membranes in skin and airways, inflammation, and spread of influenza with complaints of dry or stuffy air.^8^, ^9^, ^42^ Literature review studies have linked high or low RH with the potential development of illnesses such as asthma, and dry and irritated mucous membranes of the eyes and airways, via direct and indirect pathways of impact.^7^, ^43^, ^44^ They refer to field studies that have illustrated the complexity of these relationships and suggested different mechanisms. For asthma in particular, a population control study found an association between VOC levels and reports of asthma in children^45^ while a similar study in the UK found that asthma levels in children were significantly correlated with dampness but not with total VOCs. In the latter study, only the presence of formaldehyde in combination with damp conditions was associated with wheezing.^46^ Previous studies (mainly simulated in the laboratory settings) have linked high or low RH with the potential development of illnesses such as asthma, sick building syndrome, and dry and irritated mucous membranes of the eyes and airways, via direct and indirect pathways of impact.^7^, ^43^, ^44^ Additionally, studies have linked low RH to increased rates of infection, illness, irritation, and fatigue associated with desiccation of the precorneal tear film in the eyes, decreased mental efficiency associated with dehydration, and indirect effects via influenza viability.^7^, ^8^, ^18^ Finally, and perhaps most directly related to our findings, studies have found dry air conditions linked to increased production of the stress‐related hormone cortisol in the skin^15^ with implications for immune system responses to both dermatological, epidermal barrier and systemic inflammatory and cancer development. Any of these conditions could conceivably contribute to the increased level of stress response observed in our study. However, as discussed earlier, the mechanisms are complex and additional studies are required to demonstrate causal link and to understand underlying mechanisms.

We also found that dry conditions and humid conditions correlated with stress response to a similar degree. While physiological mechanisms are complex, and we cannot explore them given our exploratory study design, there is plausible explanation for a potential relationship in the literature. The association between dry conditions and stress responses could be explained by dry ambient air increasing the likelihood of water loss through the skin^47^, ^48^, ^49^ leading to lower skin perfusion and consequently higher cardiac activity to accommodate the water loss.^39^, ^50^ Similarly, in humid conditions, the amount of water evaporation from the skin (the mechanism of action for cooling down the body) leads to an altered thermoregulatory mechanism and, thus, increased cardiac activity.^39^, ^50^ These studies align well with our correlational analysis which suggests the potential for an optimal range in the relationship between RH and stress response. We do not presume that a specific range can be inferred from this study, nor would we minimize the potential risks associated with controlling low RH in buildings. However, our data do support further exploration through a more rigorously controlled study to test whether the range established in ASHRAE 55‐1989, or an even narrower range, would reduce physiological stress responses in individuals exposed to RH outside that range.

We did not observe a direct correlation between RH and sleep quality or a consistent relationship between RH and physical activity. However, we used structural equation models (SEM) to investigate any indirect relationships between RH and these health metrics. Our SEM results revealed a relationship between RH at the office and sleep quality mediated by stress responses at the office. This finding supports previous research that stress during work hours may be associated with poorer sleep quality.^51^ By influencing one's stress response, RH may magnify this association. Our SEM did not identify physical activity as a significant mediator of the influence of RH on stress responses or objectively measured sleep quality (Figure S7). Our findings also complement other research that suggests office workers in the US exhibit generally sedentary behavior.^52^

Because RH varies with T and absolute humidity, we expected that T might have some influence on our findings and explored its effects in several ways. Interestingly, we found T had a limited impact on the association between RH and stress responses. This may be due to the well‐controlled T in the study locations which resulted in low fluctuation of T in office settings (Figure S9). However, when we added seasonally defined T ranges to our criteria for “discomfort” (Figure S2), we found a 14% difference in stress response between discomfort and comfort groups (14%, *P *= .007, ES = 0.48). Thus, incorporating T into the criteria for discomfort reduces the difference between groups as compared to when they are based on RH alone (22%, *P *= .001, ES = 0.64). Additionally, we did not find any statistically significant relationship between temperature and participants’ stress responses. This suggests that RH has a bigger influence on stress response in our data than T. T is the primary focus of thermal comfort standards today which is understandable since humans have no RH receptor and only perceive changes in RH indirectly. Frequently, humans confuse the symptoms of changes in RH, especially to low RH, for other conditions like stuffy air, irritation of skin and eyes, or fatigue.^48^ Previous studies also indicate that high RH at surfaces is associated with growth of various indoor contaminants including mold and mildew.^53^ Our findings suggest that relative humidity may have a place in building standards beyond these traditional associations with thermal comfort and control of biological contaminants.

There is ongoing debate about the relationship between indoor RH and indoor pollutants like PM and VOCs, and the perceptions of indoor air quality and health outcomes of building occupants.^6^, ^7^, ^8^ High or low RH in buildings can be related to high occupancy or issues with the design and operation of the building environmental control systems (ECS). Unfortunately, we do not have detailed occupancy or ECS operational data for the spaces in our study. We do know that our measurements related to open office areas in four buildings had properly functioning HVAC systems designed to meet ASHRAE 62.1. Moreover, there were no significant complaints of poor air quality in any of the locations. In general, where high or low RH exists as a result of these potential confounds, there is often also an accumulation of indoor pollutants such as CO2, PM, and VOCs. We did measure levels for CO2 and PM and have used as proxies for ventilation effectiveness, high occupancy, and building ECS performance. We found them to be within expected ranges for a commercial facility (Figures S17, S18). While we do see variation in CO2 and PM levels between our discomfort and comfort RH groupings, they were not statistically significant (Figures S10, S14). As might be expected, average PM was progressively lower across the narrower RH groupings of dry, comfort, and humid (Figure S10). However, the differences were not statistically significant, and, despite the differences in PM between them, the measured stress responses between dry and humid groupings were minimal. In the narrower “humid” RH grouping, average CO2 levels were significantly lower than in the dry or comfort RH grouping (Figure S14). Based on these findings, we feel that crowding or poor ventilation is unlikely reasons for the differences in stress responses. Furthermore, we adjusted our structural equation models to test whether CO2 or PM has direct or moderating effects on stress responses. We found no significant direct effects (Figure S7) or moderating effects (Figures S8, S9) for PM, and we found no direct effects (Figure S11) or moderating effects (Figures S12, S13) for CO2. We believe these findings make confounding effects associated with ventilation, crowding, or the presence of elevated PM unlikely.

We see some correlation between calendar season and the dry and humid conditions experienced by our study participants. Seasons could potentially relate to changes in stress response due to factors such as dramatic transitions in thermal conditions between indoor and outdoor settings, physical inactivity during unpleasant weather, and effects of thermal conditioning operations on individual responses. While we were not able to address seasonal effects directly, we did explore the possible relationship indirectly. We compared stress response between participants grouped by participation during a heating or cooling season, as defined by the building operator (Figure S4). We found the difference in stress response to be only 6% (6%, *P *= .275, ES = 0.21) compared to 22% between comfort and discomfort groups considering RH alone. Furthermore, our SEM results do not suggest that any changes in physical activity, if in fact driven by season, mediate the relationship between RH and stress response. While neither of these observations rule out a seasonal effect, it seems an unlikely explanation on its own.

Finally, the findings of this study may help improve the design and operation of buildings to enhance office worker wellbeing via management of RH and behavioral interventions. Radiant heating and cooling strategies could spur energy savings by enabling a wider range of indoor air temperatures, adiabatic humidification during heating operations,^54^ and desiccant dehumidification during cooling operations.^55^ A wider range of indoor air operating temperatures within a given building could allow this strategy to save energy and also improve comfort for those that current strategies leave most dissatisfied if occupants are given the freedom to choose their workspace based on comfort or have access to personal comfort devices.^56^ Distributed sensor networks similar to those employed in our study could be implemented in workplaces and prompt individuals to take restorative actions, such as micro breaks, that reduce fatigue and irritation to the human eye caused by dry air. These networks could also help building operators improve thermal comfort through enhanced psychrometric controls. These strategies are not new but they have been narrowly applied in practice. Even when properly designed these strategies are often ineffectively executed in the field because while building operators have developed an intuitive understanding of control systems based on operating temperature set points, they lack an intuitive understanding of how to control conditioning based on mean radiant temperature or air velocity.^57^ We recommend direct testing of these strategies to measure their effectiveness in addressing the health outcomes mentioned in this paper**.**

## 5 LIMITATIONS

There are several limitations that could affect our results. First, the number of participants with sufficient data was not enough to draw a strong conclusion in the subgroup analysis (eg, to make comparisons between dry and humid zones) nor a precise cut point for RH in the correlational analysis. Since physical location was not directly monitored, we relied on physical logs and other inputs that were not always maintained by the participants. This caused a loss of data points and/or participants. Second, it would have been valuable to consider the effect of RH in the context of other environmental factors such as CO2, sound, and T, but the reduced dataset did not allow for statistically meaningful combined analysis with multiple other indoor environmental factors. Third, we did not directly consider seasonal and climate effects. Importantly, most dry‐air conditions were associated with participants in heated office buildings and RH could be a marker for other conditions associated with season or heating operations. These might include things like dry air connected with heating operations or sedentary behavior associated with inclement or uncomfortable outdoor conditions. Fourth, while the recording period was significantly longer than prior studies, many of which were largely limited to laboratory conditions, three consecutive days may not be sufficient to relate RH with long‐term stress, physical activity, and sleep. Fifth, the environmental sensors used to measure the ambient RH may not represent the exact body surface RH, and thus, we are unable to examine the effect of clothing in this study. Sixth, because of the design of our study, we cannot conclude any causality between conditions and health outcomes during work hours to outcomes outside of work hours. However, because results from past studies have indicated the possibility of such carryover effects on physiological, diurnal variations,^58^, ^59^, ^60^ our data are relevant to future investigation. In this study of four well‐functioning office buildings, the indoor environment was generally were well controlled. This caused small variation in many IEQ factors, thus limiting the ability to observe significant links between some IEQ factors and physiological outcomes that might be found in other research.^5^, ^6^, ^7^, ^8^, ^9^, ^10^

Finally, Wellbuilt for Wellbeing is a cross‐sectional, exploratory study that cannot demonstrate causation. It is possible that other characteristics of the built environment, job type, work relationships, social or economic conditions, family issues, or other individual differences may have mediating effects between relative humidity, stress, and sleep. Additionally, we are unable to fully account for individual confounders such as selection bias in the consent process and coping mechanisms that may alleviate certain effects. Using the collected data, we have explored the potentially contributing or confounding effects some of these factors including temperature, CO2, PM, physical activity, season, and basic demographics and found the relationship between RH and stress to hold. Using wearable technology, a diverse cohort of participants and a wide range of inputs and unique health outcomes, our study offers a novel and important perspective to understanding the humidity‐wellbeing relationship.

## 6 CONCLUSION

We found a 25% lower stress response between individuals spending more than 50% of their time in 30%‐60% RH compared with those spending most of their time in drier air at the office. This difference is of moderate effect size and may represent clinically significant levels cumulatively, over long‐term exposure. An interpersonal study of all our noncategorized RH exposure and stress responses data suggests there may be an even narrower optimal range for RH and stress responses around 45% RH. We identified an indirect relationship between RH and objectively measured sleep quality. These findings lend new evidence to support the 30%‐60% RH range included in the outdated ASHRAE 55‐1989 thermal comfort standard. Further rigorously controlled studies are needed to provide evidence for causation and to understand the mechanism driving this relationship. The experimental manipulation of relative humidity and other conditions may be able to address some of the limitations of the current study. Cross‐sectional, exploratory studies cannot fully address the role that characteristics of the built and ambient environment or individual differences play. Future interventions may also be able to investigate the effect that different types of cooling and heating systems, such as microclimate control, have on stress and sleep.

In the near term, these findings support a range of strategies that may improve health and comfort. Such strategies include behavioral interventions such as periodic restorative breaks during the workday as well as using personal comfort devices and radiant heating and cooling strategies to address extreme low RH while simultaneously improving thermal comfort. 50 million white‐collar workers in the US spend over 20% of their time at the office.^3^, ^4^ Over many years of work, narrowly controlling RH values to avoid both overly dry and humid conditions at the office could have a potentially large impact on the health and wellbeing of those workers.

## AUTHORS CONTRIBUTION

All authors contributed to the study conception and design. CL, BG, and KC contributed to the acquisition of data. All authors contributed to the analysis and interpretation of data. JR, BG, CL, KC, HL, BN, MM, and ES drafted the first version of the manuscript. All authors critically revised the manuscript and approved the final text.
