# Supplementary material for: Wellbuilt for wellbeing: Controlling relative humidity in the workplace matters for our health
Source: Indoor Air. 2019 Nov 25;30(1):167–79. doi: 10.1111/ina.12618 (PMC6973066; doi:10.1111/ina.12618)
Supplement: Supplementary file 1 [file INA-30-167-s001.docx]

# **Supplemental Materials**

# **Supplement S1: RH and RMSSD (parasympathetic activity)**

When comparisons between groups were done using RMSSD (an indicator of parasympathetic activities), participants in the comfort-humidity zone at the office had higher RMSSD values both at the office and outside the office where the between groups’ differences remained statistically significant. These stress response values are presented as 100msec – SDNN to simplify the visual representation of higher stress.


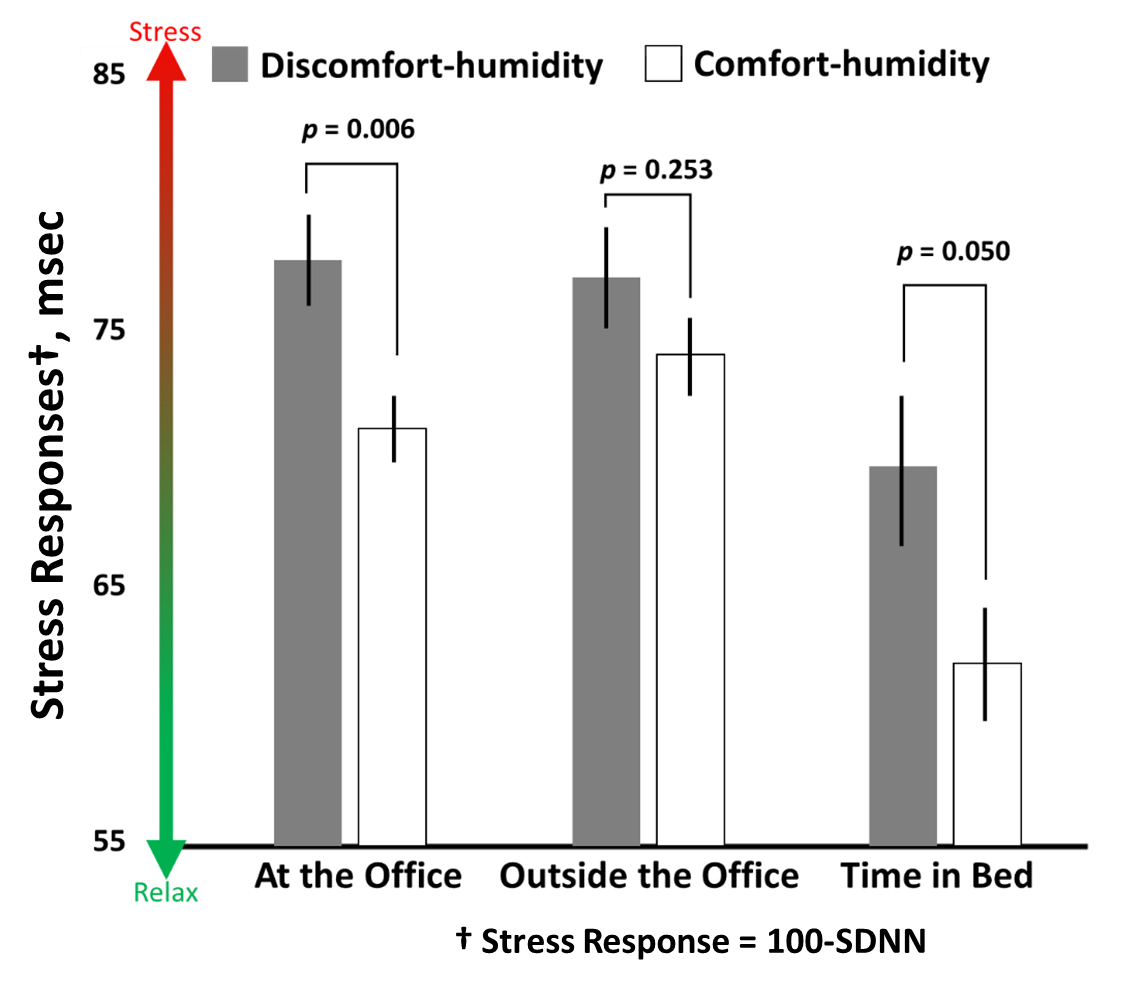


Supplement Figure S1: Group comparisons of stress responses using RMSSD (parasympathetic activity)

# **Supplement S2:** **Thermal comfort and stress responses (SDNN and RMSSD)**

To consider seasonal effects, the benchmarks for the discomfort group were adjusted based on a model of perceived thermal comfort for the built environment in each season.^1,2^ This model defined two groups: thermal comfort and thermal discomfort defined below:

● Summer season: 30% < RH < 60% and 24^°^C < T < 27^°^C

● Spring or fall season: 30% < RH < 60% and 21^°^C < T < 27^°^C

● Winter season: 30% < RH < 60% and 21^°^C < T < 25^°^C

Those in the thermal-comfort group had significantly lower stress response (SDNN) and higher relaxation (RMSSD) than those in the thermal-discomfort group. These stress response values are presented as 100msec – SDNN or 100msec – RMSSD to simplify the visual representation of higher stress.


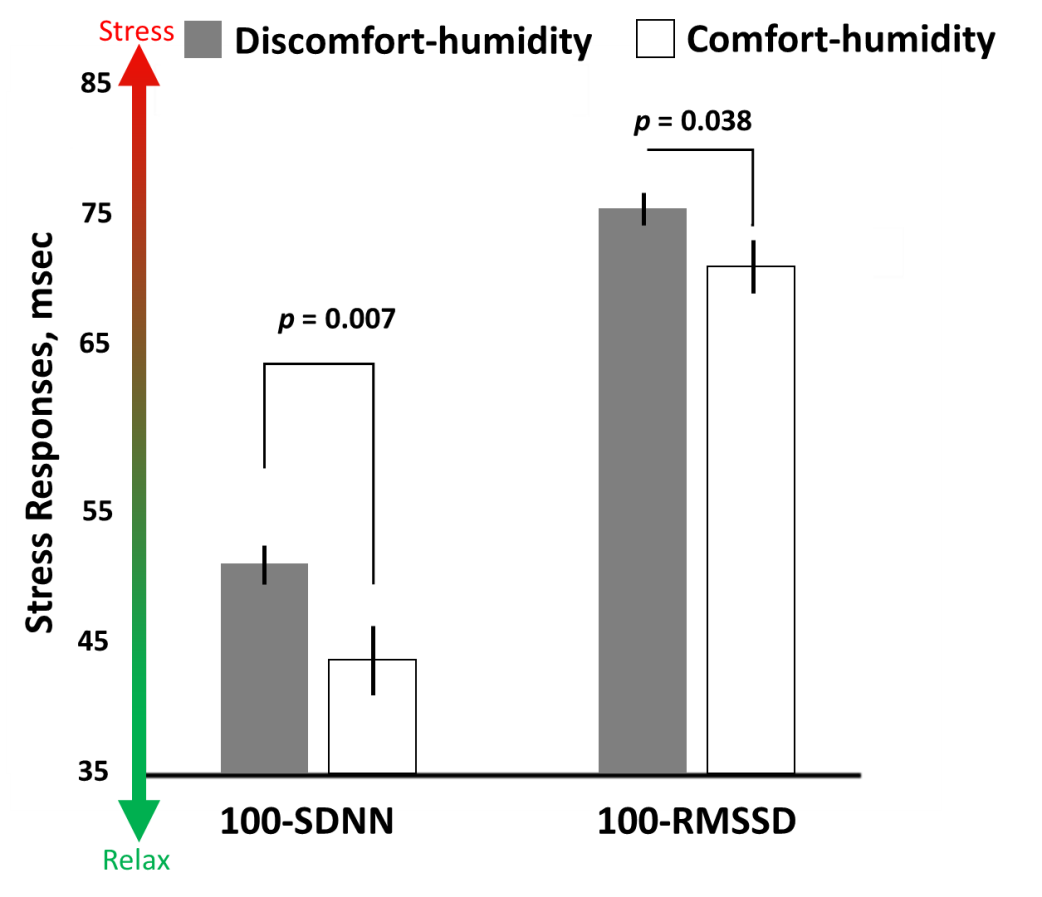


Supplement Figure S2: Thermal comfort and stress response (SDNN and RMSSD)

# **Supplement S3:** **Heating vs. cooling season and stress responses (SDNN)**

Comparison of SDNN between groups associated with measurements taken while the study building was in heating mode vs cooling mode. Building operations cannot account for all the variability between comfort and discomfort humidity groupings. Additionally, building managers reported no known issues with building systems during the study. These stress response values are presented as 100msec – SDNN to simplify the visual representation of higher stress.


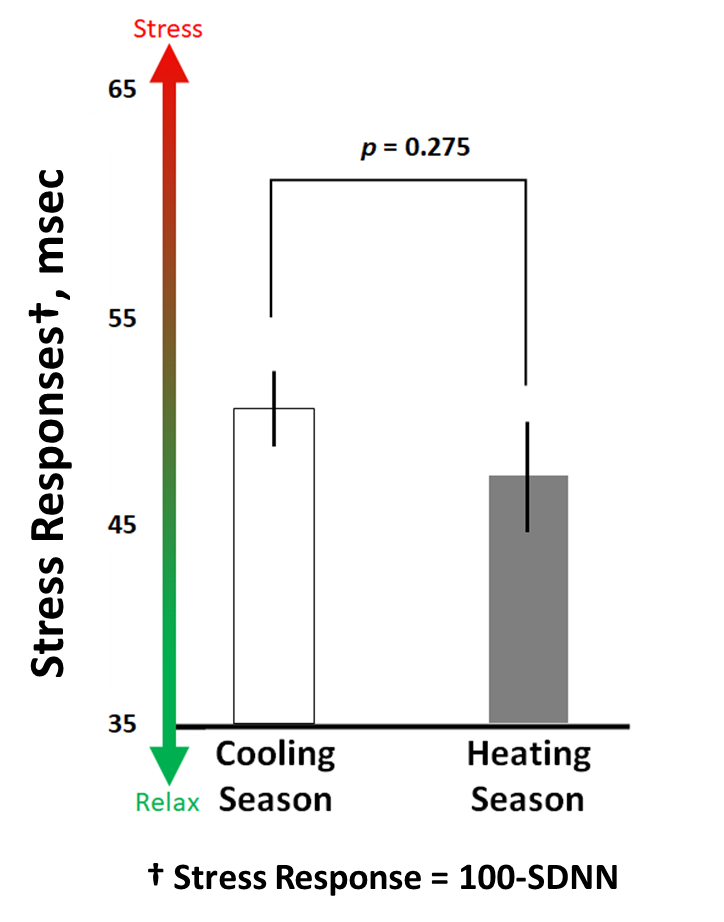


Supplement Figure S3: Heating vs. cooling season and stress responses (SDNN)

# **Supplement S4:** **Significant relationships between physical activity and RH**

While there were some significant differences in physical activity levels between the comfort and discomfort groups, the results were not consistent and were not reflected in our Structural Equation Model. During office time, both groups spent, on average, 84% of their time sitting, 9% standing, and 7% walking. There was no statistically significant difference between these profiles, nor, between overall physical activity levels measured in mG (Table 2). Results suggested there were differences in time spent sitting with those in the discomfort group sitting longer than the comfort-humidity group (Table 2). Workers in the discomfort-humidity group had 19% shorter unbroken walking bouts (*p* = 0.005, ES = 0.54) and took an average of 414 (12%) fewer steps than those in the comfort-humidity group (*p* = 0.197).

Outside the office, a higher trend of sedentary behavior was observed in the discomfort-humidity group (6%, *p* = 0.060, ES = 0.39). The difference between groups achieved statistically significant levels for moderate-to-vigorous activity, with 45% more moderate-to-vigorous activity (*p* = 0.033, ES = 0.44, Table 2) observed in the comfort-humidity group.

# **Supplement S5: Structural equation models for multivariate analysis**

In our original model, we hypothesized reciprocal relationships between physical activity, stress response and sleep quality. However, after testing this model, physical activity at the office was not a significant mediator for the relationship between RH and either sleep quality or stress responses at the office.


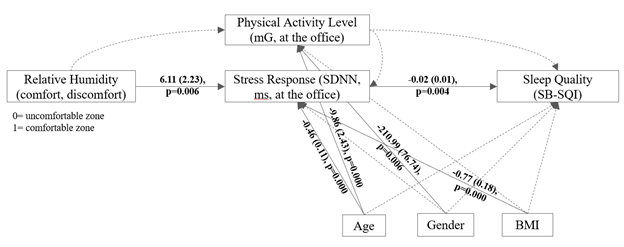


Supplement Figure S5: SEM for analyzing multivariate hypothesized relationships

# **Supplement S6 Table ST6:** **Complete SEM results**

Maximum likelihood estimation (MLE) was used to determine the significant path coefficients in the model, as well as parametric bootstrapping, a method that derives robust standard errors. Moreover, by utilizing the full information maximum likelihood (FIML) method, missing values are not replaced or imputed, but handled within the analysis model. The lavaan package in R was used for model fit and validation.

| ST6. Structural equation model results | | | |
| --- | --- | --- | --- |
| Input (Variable) | Outcome (Variable) | Coefficient estimate (SE) | 95% CI |
| Relative Humidity | Physiological stress response at the office (Mean SDNN at the office) | 6.11 (2.23)^a^ | 1.71 to 10.51 |
| Age | Physiological stress response at the office (Mean SDNN at the office) | -0.46 (0.11)^a^ | -0.68 to -0.24 |
| BMI | Physiological stress response at the office (Mean SDNN at the office) | -0.77 (0.18)^a^ | -1.13 to -0.42 |
| Age | Objective activity at the office (ActLevAvg at the office) | -9.65 (2.43)^a^ | -14.67 to -5.06 |
| Gender - Female | Objective activity at the office (ActLevAvg at the office) | -210.99 (76.74)^a^ | -362.32 to -59.67 |
| Physiological stress response at the office (Mean SDNN at the office) | Sleep quality (SB-SQI) | -0.02 (0.01)^a^ | -0.03 to -0.01 |
| Global estimation with maximum likelihood approach was used for simultaneous estimation of the path coefficients. Bootstrapping with 100 repetitions used to derive CI and standard error for estimates.  ^a^p < 0.01  ^b^p < 0.05 | | | |

We tested the quality of SEM fit using the comparative fit index (CFI) score and standardized root mean square residual (SRMR) score. CFI greater than 0.9 and SRMR less than 0.08 is considered a good fit. For our model, model fit indices were CFI = 1.00 and SRMR = 0.005. .

# **Supplement S7: Testing effects of air quality (PM) on physical activity and stress at work**

As an air quality measure that may be a potential confounder, we tested if particulate matter (PM) was directly related to physical activity or stress at work using the SEM model represented by Supplement Figure S7. The model indicates that our data does not show significant relationship between PM and physical activity or stress at work.


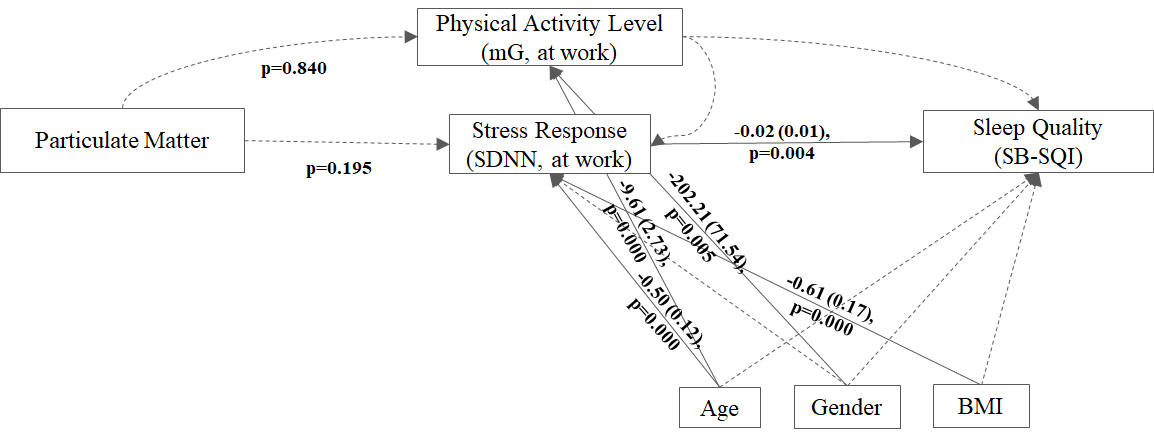


Supplement Figure S7: Model with direct effects of PM on physical activity and stress at work

# **Supplement S8: Testing moderating effects of air quality (PM) on the effects of RH**

To understand if PM moderates the effect of RH on stress at work, we fit the SEM model represented by Supplement Figure S8. We did not identify significant moderating effects of PM on the relationship between RH and stress at work for our data.
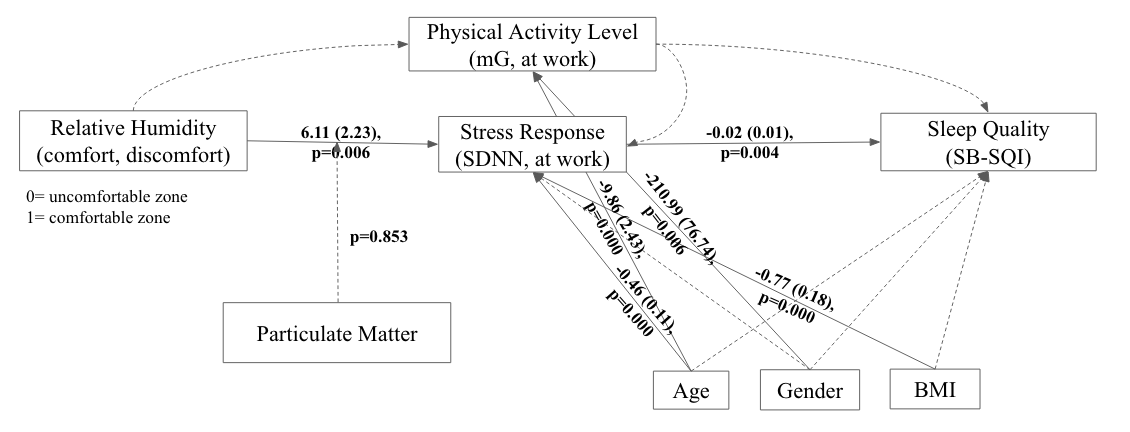


Supplement Figure S8: Model with moderating effect of PM on the effects of RH

# **Supplement S9: Testing indirect effects of air quality (PM) via RH**

We also tested if PM had an indirect effect on physical activity and stress at work via RH using the SEM model represented by Supplement Figure S9. We did not identify significant indirect effects of PM via RH in our data.


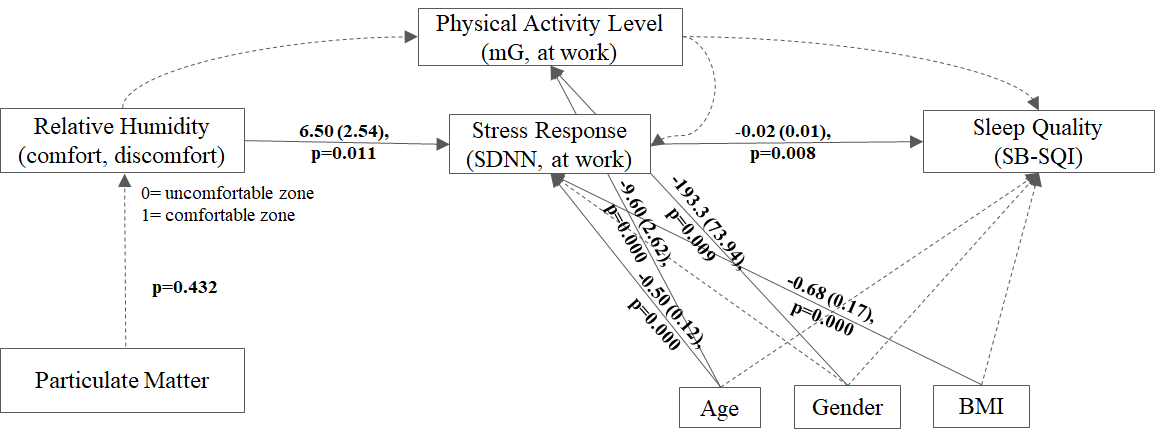


Supplement Figure S9: Model evaluating indirect influence of PM via RH.

# **Supplement S10: Variation of PM by RH grouping**

The PM varies across the three different RH categories were compared by analysis of variance (ANOVA).

**
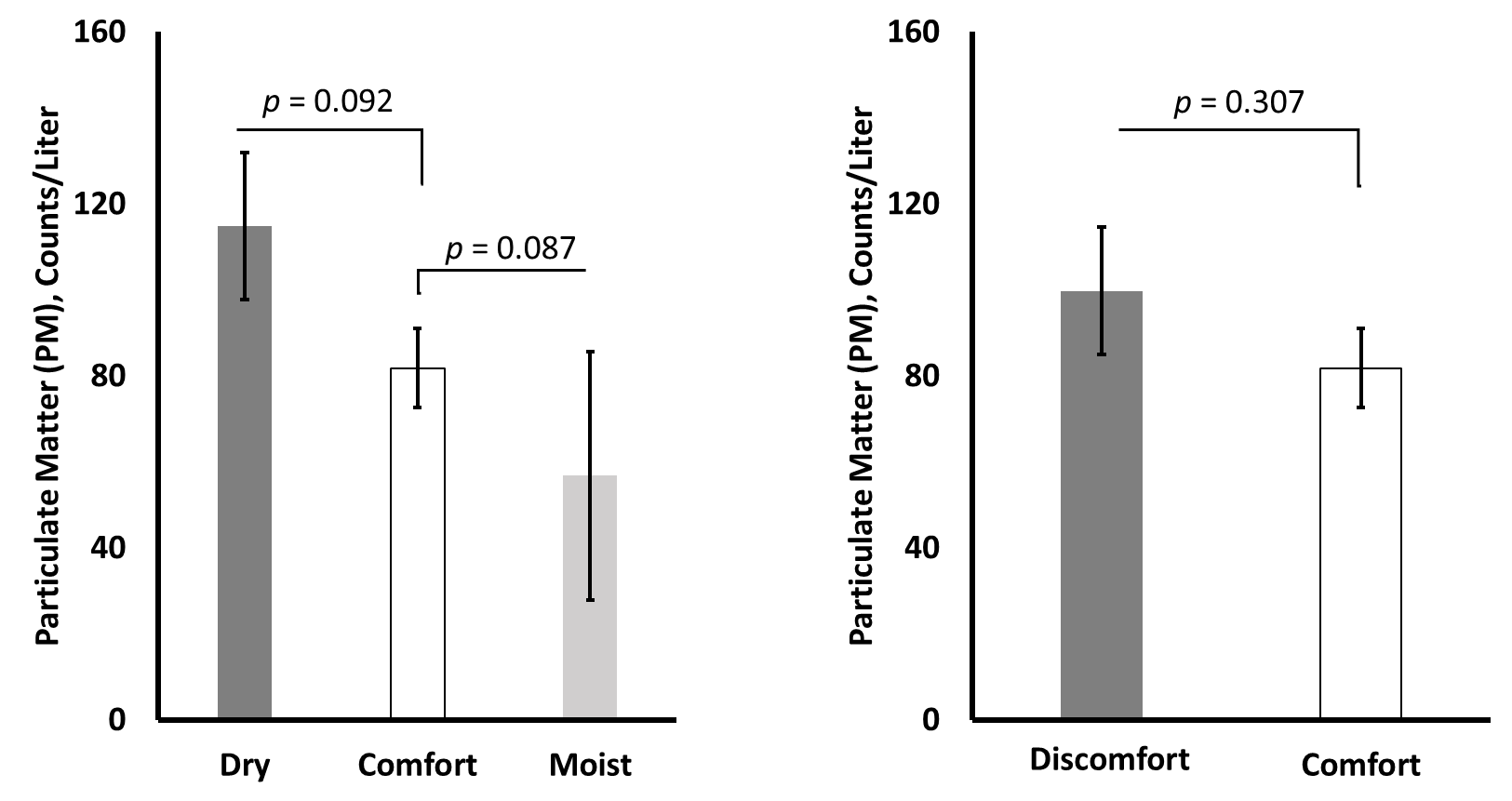
**

Supplement Figure S10: Variation of PM by RH comfort grouping

#

# **Supplement S11: Testing effects of air quality (CO2) on physical activity and stress at work**

As an air quality measure that may be a potential confounder, we tested if carbon dioxide (CO2) was directly related to physical activity or stress at work using the SEM model represented by Supplement Figure S11. The model indicates that our data does not show significant relationship between CO2 and stress at work but there is a significant relationship between CO2 and physical activity (p-value < 0.05).


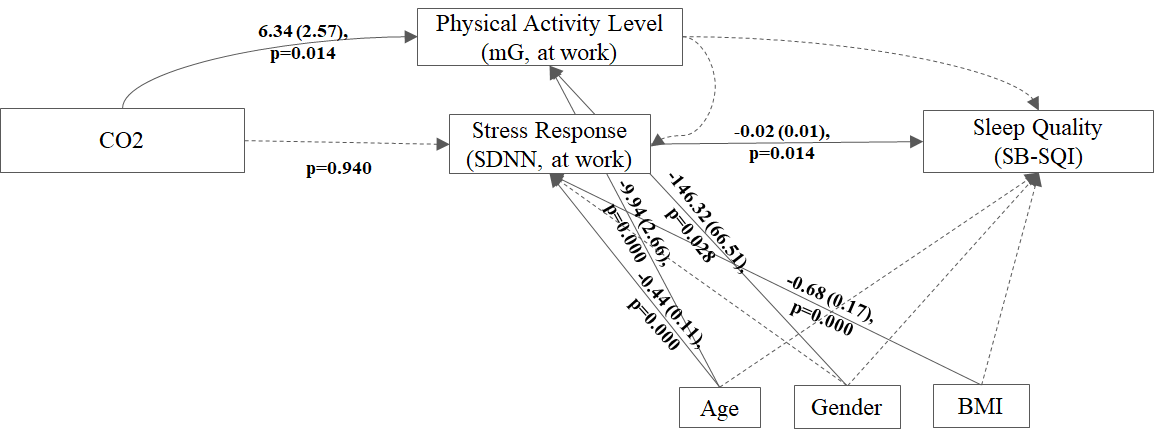


Supplement Figure S11: Model with the direct effects of CO2 on physical activity and stress at work

# **Supplement S12: Testing moderating effect of air quality (CO2) on the relationship between RH and stress at work**

To understand if CO2 moderates the effect of RH on stress at work, we fit the SEM model represented by Supplement Figure S12. We found that there is no moderating effect of CO2 on the relationship between RH and stress at work in our data.


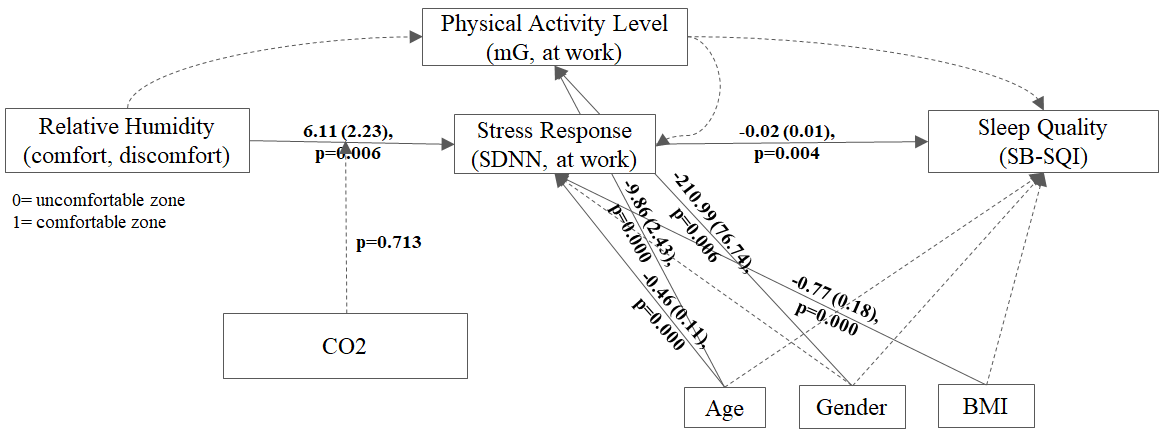


Supplement Figure S12: Model with CO2 moderating the relationship between RH and stress at work

# **Supplement S13: Testing indirect effects of air quality (CO2) via RH**

We also tested if CO2 had an indirect effect on physical activity and stress work via RH using the SEM model represented by Supplement Figure S3. We did not identify significant indirect effects of CO2 via RH in our data.


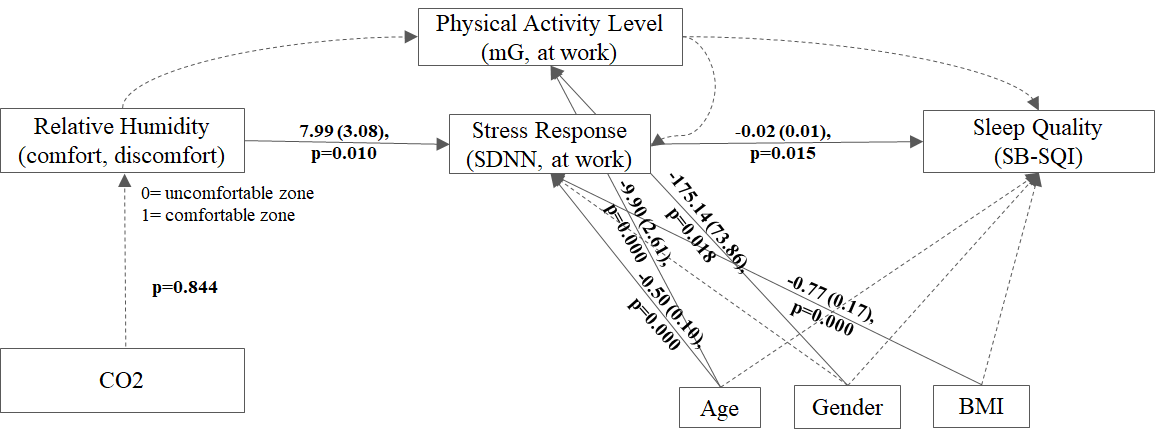


Supplement Figure S13: Model evaluating the indirect influence of CO2 via RH

# **Supplement S14: Variation of CO2 by RH grouping**

We evaluated how CO2 varied by RH grouping and found no statistically significant difference between the discomfort and comfort RH groupings.


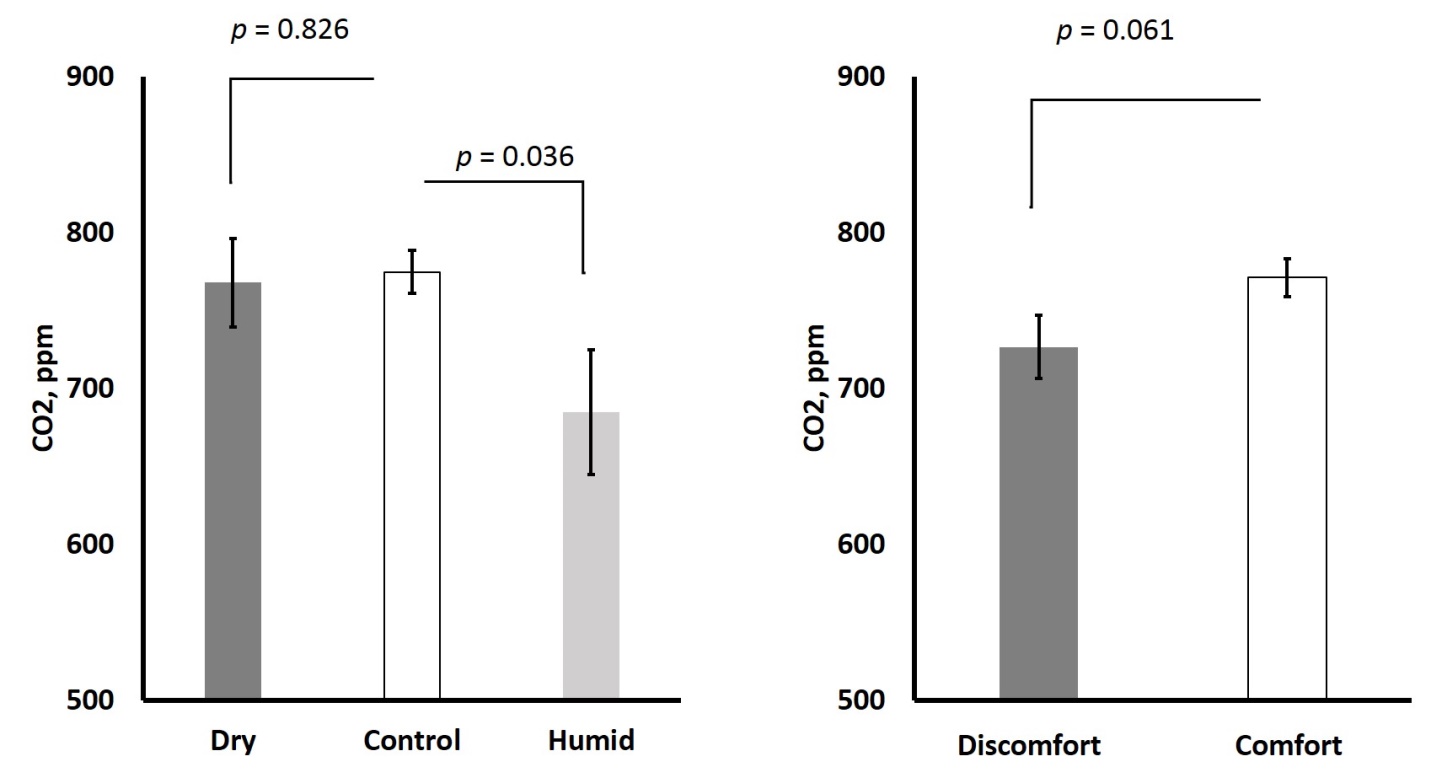


Supplement Figure S14: Variation of CO2 by RH comfort grouping

#

# **Supplement S15:** **RH variation across seasons, study locations, and space types**

Probability Distribution plots visualize RH measured at each site during data collection. No data was recorded when no participants were run: Site B during building heating season, Site C during building heating and shoulder seasons, and Site D during building cooling season. RH sensor range was 10-90%RH, resolution 0.3%, accuracy +/- 4%. Kernel bandwidth set to 0.2 in order to smooth density function.


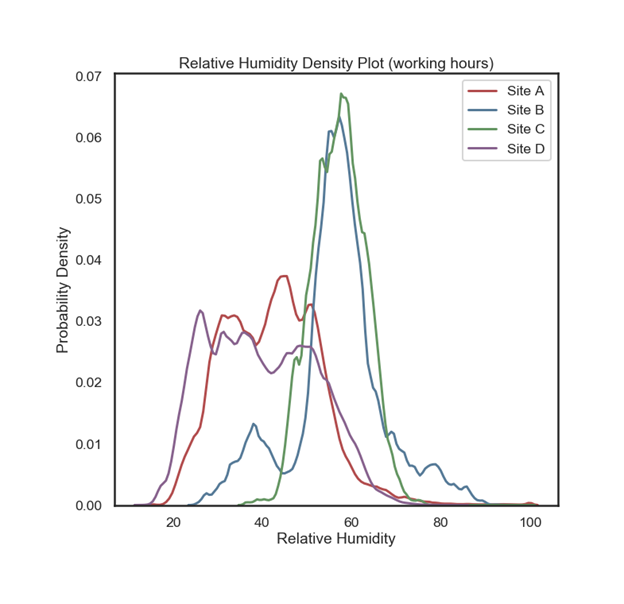


Supplement Figure S15: Probability distribution plot for RH

# **Supplement S16: Temperature variation across study locations**

Probability distribution plots visualize temperature (T) measured at each site during data collection. No data was recorded when no participants were run: Site B during building heating season, Site C during building heating and shoulder seasons, and Site D during building cooling season. T sensor range was 0-40C, resolution .2C, accuracy +/- 1C. Kernel bandwidth set to 0.2 in order to smooth density function.


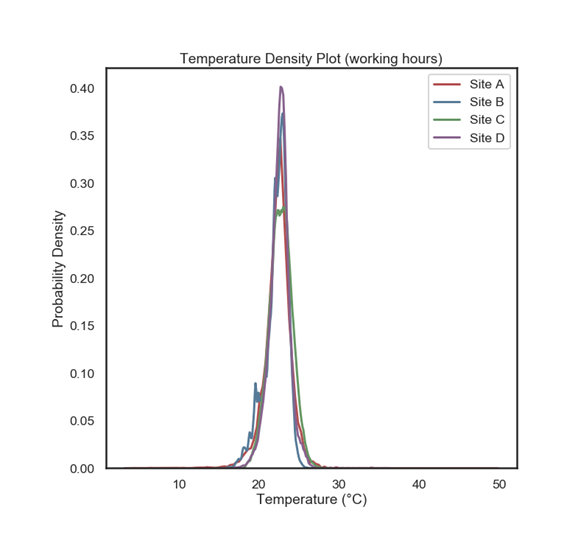


Supplement Figure S16: Probability distribution plots for temperature.

#

# **Supplement S17:** **CO2 variation across seasons, study locations and space types**

Probability Distribution plots visualize RH measured at each site during data collection. No data was recorded when no participants were run: Site B during building heating season, Site C during building heating and shoulder seasons, and Site D during building cooling season. CO2 sensor range was 0-2,000ppm, resolution 10ppm, accuracy +/- 50ppm. Kernel bandwidth set to 0.2 in order to smooth density function.


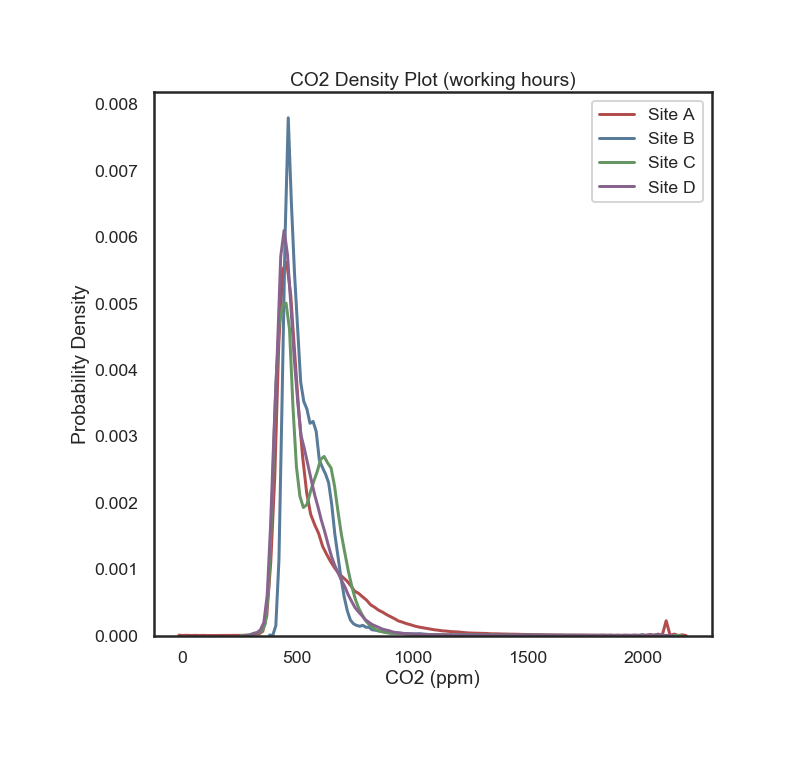


Supplement Figure S17: Probability distribution plot for CO2

# **Supplement S18:** **Particulate Matter variation across locations**

Probability distribution plots visualize PM measured at each site during data collection. No data was recorded when no participants were run: Site B during building heating season, Site C during building heating and shoulder seasons, and Site D during building cooling season. Particulate matter measurements were made with an optical sensor that detects light scattered from particles as they pass through a light source. The sensor counts particles larger than 1 micron diameter (PM1+ in the chart below). There is no "community standard" method to label the PM measured from the sensor in this study. The sensor performance has been compared to PM2.5 and PM10 measured using a reference instrument and was found to correlate most strongly with coarse particle mass (between 2.5 and 10 microns). In this study, all particulate matter data is referred to as PM.  The sensor has a range of 0-28,000 counts per liter, a resolution of 250 counts per liter, and an accuracy of 250 counts per liter +/- 20%.


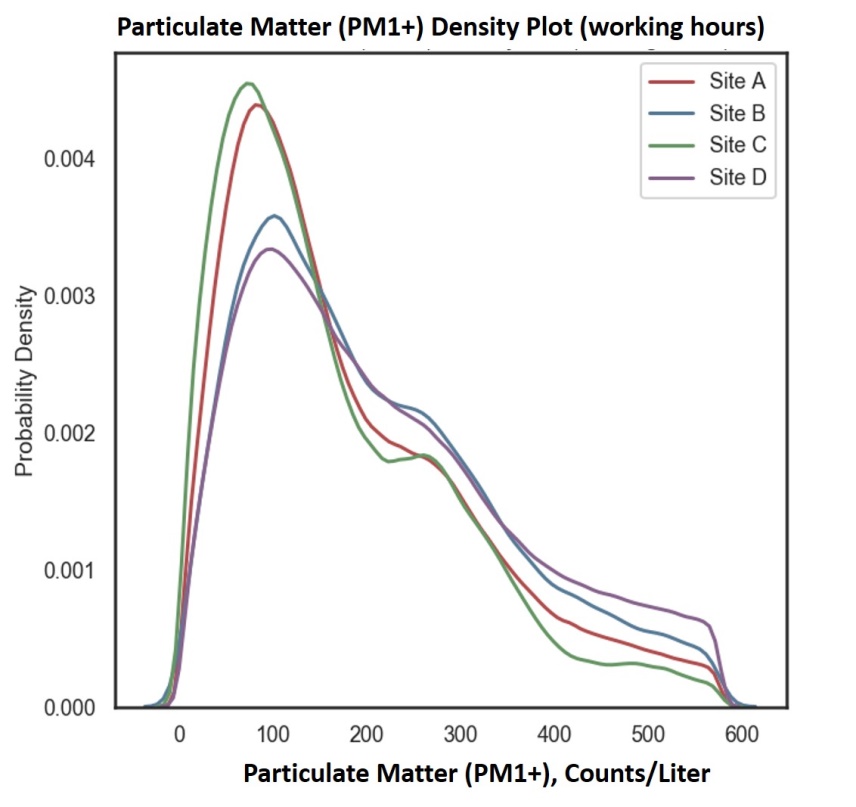


Supplement Figure S18: Probability distribution plot for Particulate Matter

**References**

1. Blume F, Hudak J, Dresler T, et al. NIRS-based neurofeedback training in a virtual reality classroom for children with attention-deficit/hyperactivity disorder: study protocol for a randomized controlled trial. *Trials.* 2017;18(1):41.

2. Variability HR. Standards of measurement, physiological interpretation, and clinical use. Task Force of the European Society of Cardiology and the North American Society of Pacing and Electrophysiology. *Circulation.* 1996;93(5):1043-1065.
